# Monitoring of cryptosporidiosis: a shift in circulating *Cryptosporidium hominis* subtypes, the Netherlands, 2016 to 2024

**DOI:** 10.2807/1560-7917.ES.2026.31.36.2600037

**Published:** 2026-09-10

**Authors:** Thomas J Roodsant, Sietze Brandes, Alewijn Ott, Ingrid HM Friesema, Roan Pijnacker, Jorrit J Hofstra

**Affiliations:** 1 Centre for Infectious Disease Control, National Institute for Public Health and the Environment, Bilthoven, The Netherlands; 2 Certe Laboratory for Infectious Diseases, Groningen, Netherlands

**Keywords:** Cryptosporidium, C. hominis, C. parvum, gp60, cryptosporidiosis, Cryptosporidium, epidemiology, food-borne infections, laboratory surveillance, molecular methods, protozoal infections, The Netherlands, waterborne infections, zoonotic infections

## Abstract

**BACKGROUND:**

Protozoan parasites *Cryptosporidium* spp. are important causes of gastrointestinal disease. They can cause large outbreaks. In the Netherlands, cryptosporidiosis is not notifiable, and monitoring relies on voluntary laboratory reporting.

**AIM:**

We aimed to describe the epidemiology of human cryptosporidiosis in the Netherlands from 2016 to 2024 and investigate associations with subtype and meteorological variables.

**METHODS:**

Three medical microbiology laboratories submitted faecal or DNA samples from patients with laboratory-confirmed cryptosporidiosis, with information about age and sex, for species identification and optional *gp60* subtyping. Associations between numbers of infected patients and *Cryptosporidium* species, *gp60* subtype, temperature and precipitation were analysed.

**RESULTS:**

In 2016–2024, 2,500 patients had laboratory-confirmed cryptosporidiosis, with a marked peak in 2023 (n = 622) caused by *C. hominis*. The lowest number of *Cryptosporidium* infections was recorded in 2021 (n = 61), with no *C. hominis*. Among all age groups, children aged ≤ 4 years had the highest number of infections (n = 493; 27%). In total, 1,166 (55%) patients had *C. hominis* and 951 (45%) had *C. parvum* infections. Infections with *C. hominis* peaked in September. We observed a weak association between temperature and the number of patients with *C. hominis*. Of the *C. hominis* identified*,* 207 were *gp60* typed and an increase in subtype diversity was observed since 2022.

**CONCLUSION:**

In the Netherlands, *C. hominis* drives the annual fluctuations in cryptosporidiosis. Continuous surveillance, including information about exposure to risk factors and molecular typing, is key to a better understanding of the underlying causes of *Cryptosporidium* spp. infections.

**Key public health message**

**What did you want to address in this study and why?**

*Cryptosporidium* are parasites that can cause diarrhoea in humans and animals. Even ingestion of only a small number of parasite cells can lead to an infection. We aimed to study infections with *Cryptosporidium* in the Netherlands between 2016 and 2024 by analysing data from three microbiology laboratories testing samples from patients with confirmed *Cryptosporidium* infections.

**What have we learnt from this study?**

In 2016–2024, 2,500 people were diagnosed with *Cryptosporidium* infection. Almost 500 of them were children aged 4 years and younger. The highest number of infections was diagnosed in 2023 (n = 622) and lowest in 2021 (n = 63). *Cryptosporidium hominis*, which is only transmitted between humans, caused 55% of the infections. Weather variables could not explain the annual fluctuations in cases.

**What are the implications of your findings for public health?**

The annual fluctuation in *Cryptosporidium* infections in the Netherlands is driven by the number of *C. hominis* infections. Subtyping of *C. hominis* from patient samples showed that *C. hominis* diversity has increased but did not explain the annual fluctuations. Other factors, such as immunity, human behaviour or virulence genes may drive the fluctuations. Identifying these factors is essential for developing public health interventions.

## Introduction

*Cryptosporidium* spp. are protozoan parasites with a wide host range that can cause gastrointestinal disease, resulting in diarrhoea in many vertebrates, including humans. In most people, cryptosporidiosis is generally self-limiting but can be severe in people with weakened immune systems [1]. After clearing the *Cryptosporidium* spp. infection, gastrointestinal sequelae are frequently reported [2]. Human infections are predominantly caused by *C. hominis* and *C. parvum* [3]. While *C. parvum* has a broad host range including livestock such as cattle, *C. hominis* is almost exclusively found in humans [3]. *Cryptosporidium* spp. are transmitted as oocysts via the faecal-oral route, either directly from person-to-person, via contaminated food or drinking and recreational water [3]. After an incubation period of 2–10 days (average: 7 days), an infected host can shed over a billion oocysts per day, while the infective dose is as low as 10–30 oocysts [4]. The oocysts can survive for months in the environment and are extremely chlorine tolerant [3].

In the 2021 annual report on cryptosporidiosis within the European Union (EU), the notification rate was 1.8 confirmed cases per 100,000 inhabitants and ranged from < 1 to 16.7 between countries [5], although the true incidence is likely higher due to under-reporting and underdiagnosis [6]. In total, 94% of the confirmed cases in the report were from only six countries. The number of reported cases is dependent on the number of samples tested for this parasite, as several countries reported no or only a few cases. In 20 EU countries cryptosporidiosis is mandatorily notifiable, and in two countries notification is voluntary. Generally, the number of cryptosporidiosis cases shows an annual double peak pattern, especially in countries with a comprehensive surveillance system and a large livestock population, such as the United Kingdom (UK) and Ireland [5-8]. A small peak is seen in March–April, which is primarily associated with *C. parvum*, and attributed to close contact with newborn farm animals [6-9]. A larger peak is seen in August–October, primarily associated with *C. hominis,* and attributed to recreational waters and international travel [6-9]. In 2023, Denmark, Germany, Spain and the UK reported marked increases of cryptosporidiosis cases [10-13].

In the Netherlands, cryptosporidiosis is not notifiable. Thus, monitoring relies on voluntary participation of medical microbiology laboratories (MMLs). To better understand the infection dynamics, *Cryptosporidium* spp. can be characterised by species identification and by subtyping through sequencing the highly polymorphic glycoprotein 60 kDa (*gp60*) gene. The *gp60* gene encodes a surface glycoprotein involved in host cell invasion, and distinct *gp60* variants show differences in pathogenicity [14]. In the Netherlands, IbA10G2 was the dominant circulating *C. hominis* subtype that caused a peak in cryptosporidiosis in the late summer of 2012 [15,16]. A subsequent population-based case–control study, including species identification, showed that in 2013 and 2014 most infections were caused by *C. parvum,* followed by another peak of *C. hominis* infections in the late summer of 2015 [17]. During the study period, the main risk factors for cryptosporidiosis were having contact with a household member who had diarrhoea and consuming barbequed food [17]. Multiple studies have found an association between cryptosporidiosis and precipitation or temperature [18]. To better understand the causes of peaks in cryptosporidiosis in the Netherlands, we aimed to analyse data on cryptosporidiosis from 2016 to 2024. Additional *gp60* subtyping of a subset of samples and meteorological variables were added to explore potential genotypical or meteorological associations with the peaks in cryptosporidiosis cases in the Netherlands.

## Methods

### Data and sample collection

Three MMLs situated in northern, central and southern Netherlands submitted faecal or DNA samples from all their laboratory-confirmed cryptosporidiosis cases based on PCR detection to RIVM between January 2016 and December 2024. Laboratories were also asked to provide minimal patient data on sampling date, age and sex. In case sampling date was missing, date of sample receipt at the laboratory was used.

### Laboratory analyses

From faecal samples, the DNA was extracted using the QIAamp DNA Mini Kit (Qiagen, Venlo, the Netherlands) or the Maxwell RSC Fecal Microbiome DNA kit (Promega, Madison, the United States (US)) including bead beating according to the manufacturer’s protocol. Initial identification of *C. hominis* and *C. parvum* was performed using a duplex real-time PCR targeting the *C. parvum* allele of *Lib13* and the *C. hominis* alleles of *gp60* (2016–2019 [16]) or *A135* (2019–2024 [19]). Since 2020, samples that tested negative for *C. hominis* and *C. parvum* were also typed with a real-time PCR targeting the *SSU* rRNA gene, with subsequent sequencing of the amplicon [20]. For *gp60* subtyping, samples were randomly selected from all included years (2019–2024). In years with fewer than 30 *C. hominis* cases, all available samples were subtyped. In years with 30 to 100 cases, at least 20 samples were selected, and in years with more than 100 cases, at least 30 were subtyped. During years with increased diversity (2022–2024), additional samples were analysed, up to 60 per year, to ensure that subtypes with a 5% frequency would be detected with a 95% probability. The *gp60* subtyping was performed using a nested PCR with primers AL3531, AL3535, AL3532 and AL3534 as previously published [21]. Amplicons were cleaned with PUREIT EXOZAP (Ampliqon) and Sanger sequenced. Sequencing data were analysed with Cryptogenotyper v1.5 [22,23].

### Data analysis

Publicly available data on the Dutch population and the weather were retrieved from Statistics Netherlands (CBS). Data on mean weekly temperature and precipitation were obtained from the Royal Netherlands Meteorological Institute (KNMI).

Pearson’s correlation test was used to determine the relationship between mean weekly temperature and precipitation (in mm) and the number of *C. hominis* cases. The effect of temperature and precipitation on the number of *C. hominis* cases was assessed for up to 4 weeks using time-lagged variables. Analyses were done for all weeks of the year and summer only (July–September: week 27–40). The *gp60* subtypes were used to calculate the Simpson’s Diversity Index using the vegan package (2.7–2) (https://vegandevs.github.io/vegan/reference/diversity.html). We compared Simpson’s diversity between samples from 2019–2020 and 2022–2024 using a permutation test (n = 1,000). Individual subtypes were pooled and randomly reassigned into two groups of the original sizes. For each permutation, we calculated the difference in Simpson’s Diversity indexes. The p value was calculated as the proportion of permutations with an absolute difference greater than or equal to the observed difference, using the (*k* + 1)/(*n* + 1) correction, where *k* is the number of permutations meeting this criterion and *n* is the total number of permutations. Data analyses were performed in R (version 2024.12.1) (https://www.r-project.org/). Data visualisation was done using GraphPad Prism (version 10.2.2) (https://www.graphpad.com/features).

## Results

### Laboratory-confirmed patients with *Cryptosporidium*

**Figure 1:**
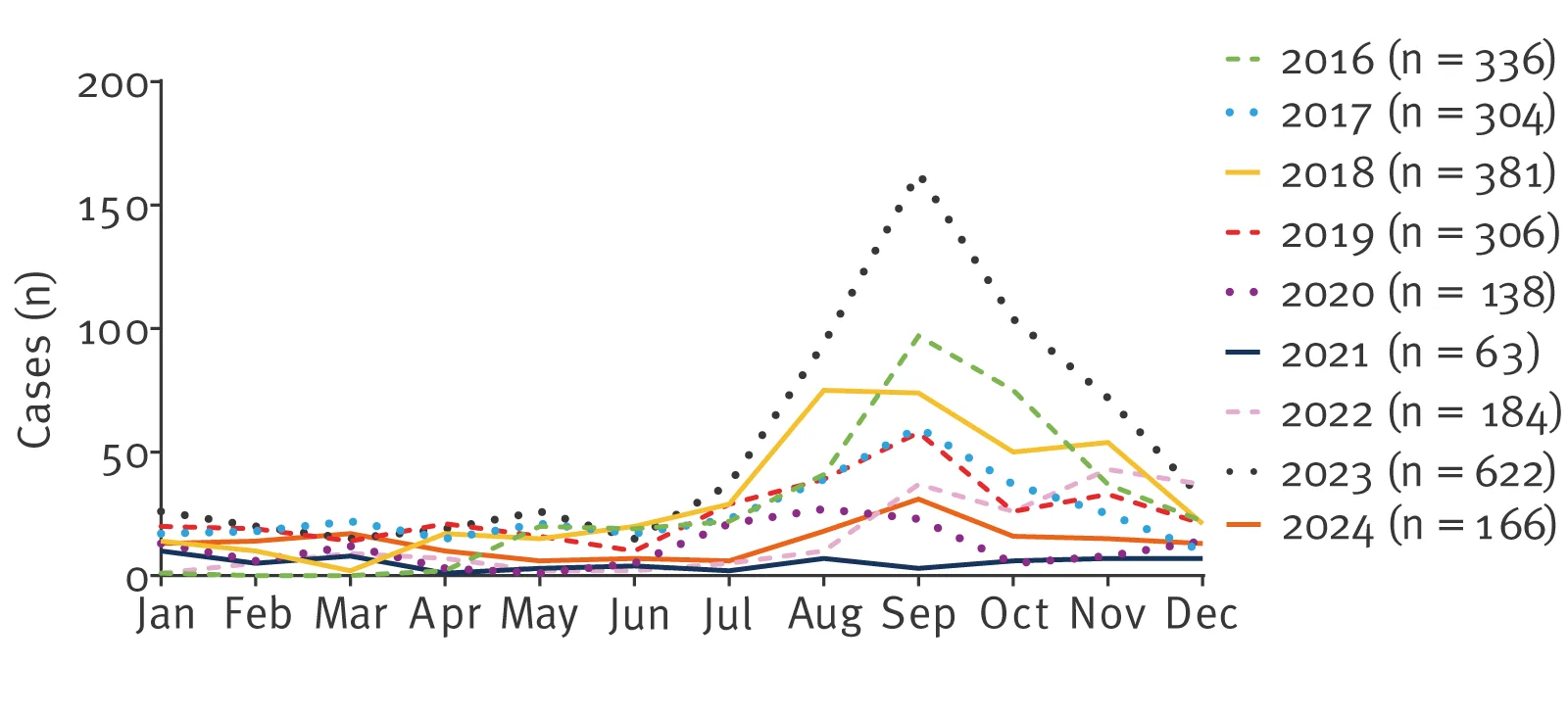
Number of patients with laboratory-confirmed cryptosporidiosis, by month, the Netherlands, 2016–2024 (n = 2,500)^a^

^a^ Data from three medical microbiology laboratories.

Between January 2016 and December 2024, three laboratories submitted data about 2,500 patients with laboratory-confirmed cryptosporidiosis. Of these patients, 1,091 (44%) were diagnosed between July and September (week 27–40). We received 2,437 (97%) *Cryptosporidium* spp. positive samples from these laboratories. The number of patients with laboratory-confirmed cryptosporidiosis varied between years, with the highest number in 2023 (n = 622) (Figure 1). In most years (2016, 2017, 2018, 2019 and 2023), a seasonal peak was observed in September (ranging from week 37 to week 39), which was most profound in 2023 with 163 infections.

### Age and sex distribution

**Figure 2:**
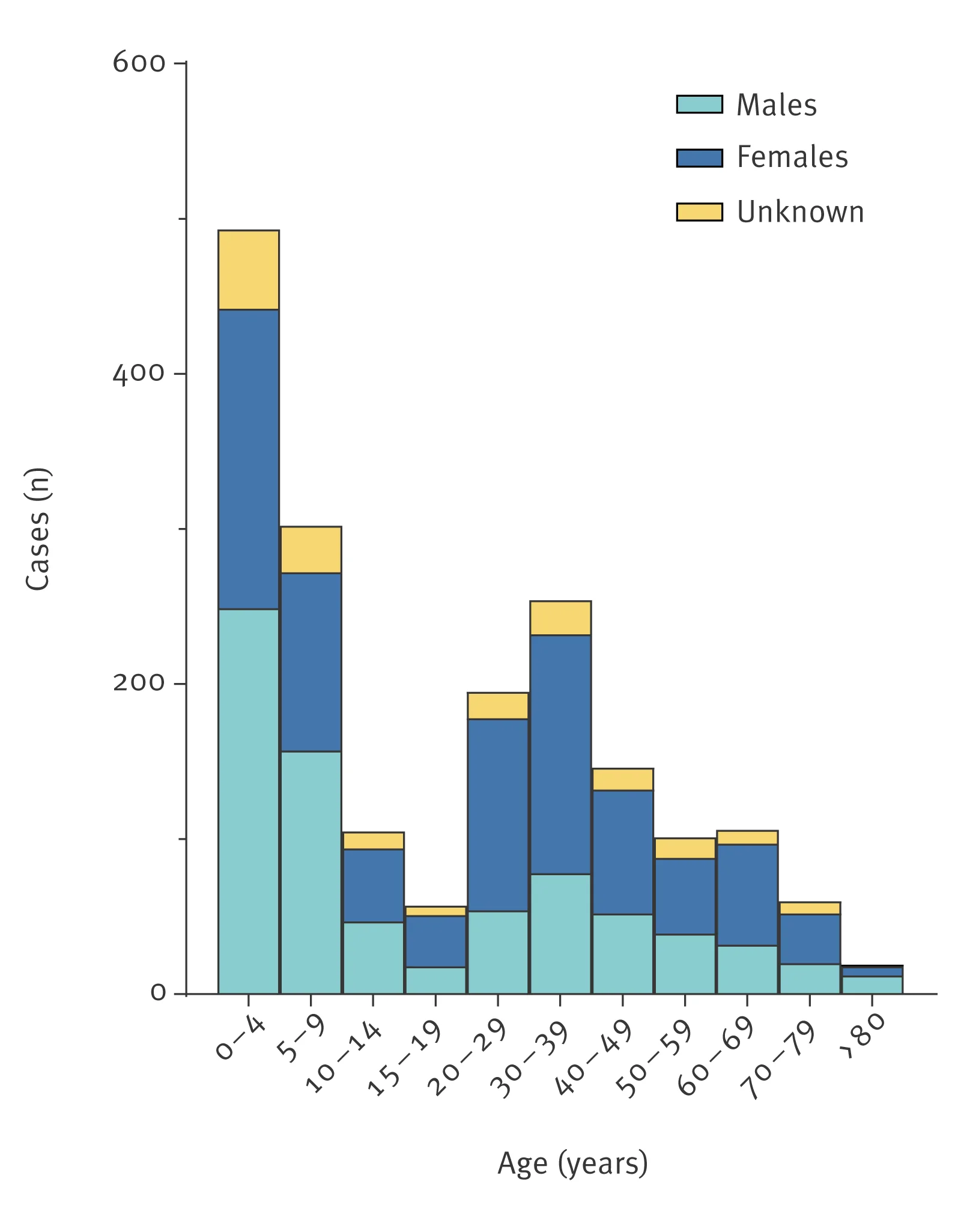
Distribution of patients with laboratory-confirmed cryptosporidiosis, by age and sex, the Netherlands, 2016–2024 (n = 1,838)^a^

^a^ Data from three medical microbiological laboratories.

For most patients, the age (n = 1,838; 74%) and/or sex (n = 1,691; 68%) was known. Overall, *Cryptosporidium* spp. infections were more common in females (n = 917; 54%) than in males (n = 774; 46%). The proportion of females was highest among the 20–29-year-olds (85/120; 71%) (Figure 2). Among all age groups, children aged ≤ 4 years had the highest number of infections with *Cryptosporidium* (n = 493; 27%), which included 25 cases in infants (aged < 1 year). The second most affected age group was the 5–9-year-olds (n = 302; 16%).

### *Cryptosporidium* species and seasonality

**Figure 3:**
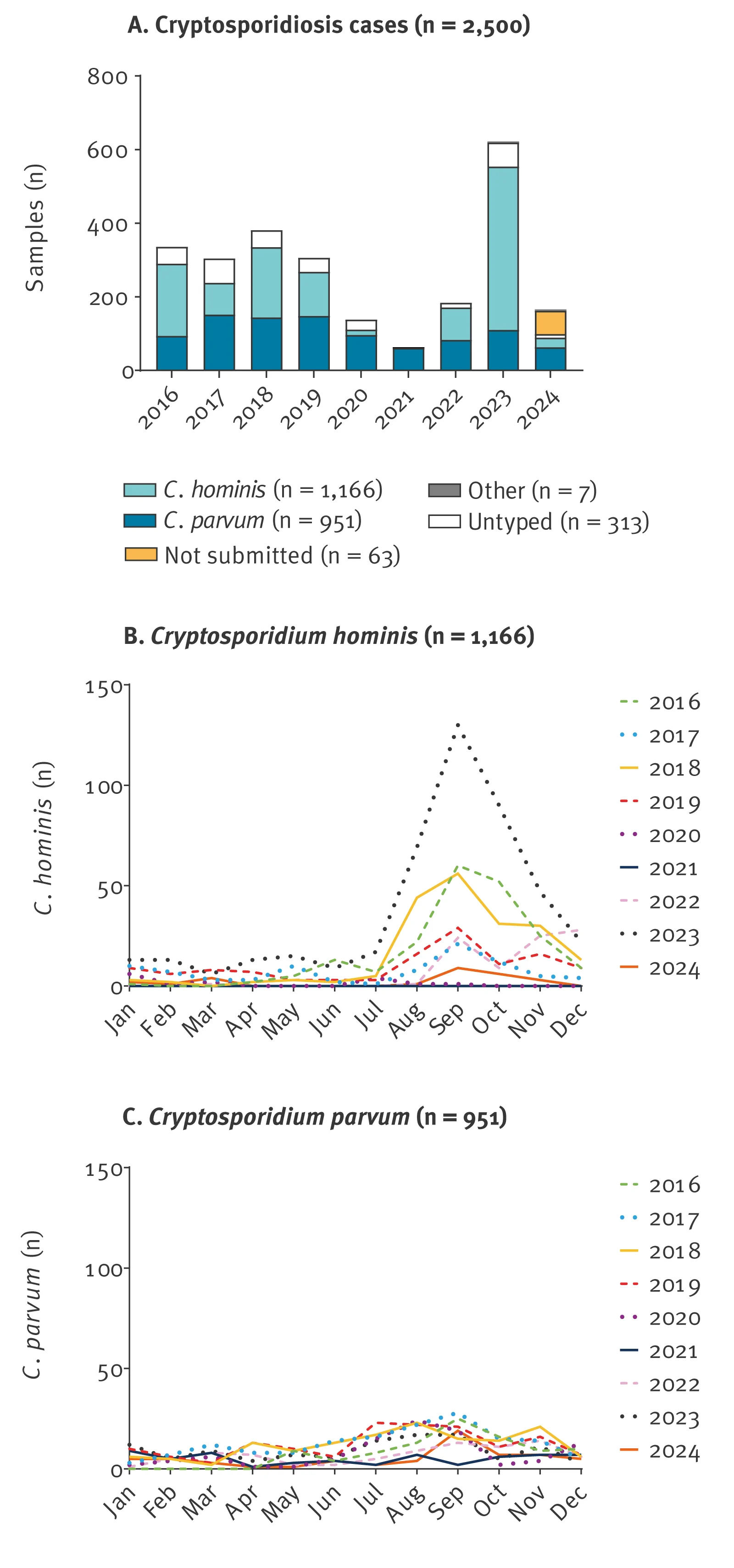
Species identification of samples from patients with laboratory-confirmed cryptosporidiosis, the Netherlands, 2016–2024^a^

^a^ Data from three medical microbiology laboratories.

The species was successfully identified in 87% (2,115/2,437) of the received samples. During the peak year of 2023, a high proportion of samples was untypable, which prompted us to change our duplex *C. hominis* and *C. parvum* PCR, change DNA extraction method to Maxwell RSC Fecal Microbiome kit and test negative samples in the 18S qPCR. We retested all untyped samples submitted since 2019 (n = 146), in which we successfully typed an additional 66 samples. In total, *C. hominis* was detected in 55% (n = 1,166) and *C. parvum* in 45% (n = 951) of *Cryptosporidium* spp. positive samples. Additionally, four cases of *C. meleagridis* and three cases of *C. ubiquitum* were identified. The annual number of samples with *C. parvum* was more stable (range: 61–152) than the annual number of samples with *C. hominis*, which differed considerably, ranging from 0 to 444 (Figure 3A). Between 2016 and 2019, 86–196 *C. hominis* samples were identified yearly, which dropped during the COVID-19 pandemic to 15 (2020) and 0 samples (2021), but increased to 88, 444 and 26 in 2022, 2023 and 2024, respectively. For *C. parvum,* the decline during the COVID-19 pandemic was more moderate (96 in 2020, 61 in 2021) than observed for *C. hominis*. Each year, the number of *C. hominis* infections was low from January until June (≤ 15 cases/month) and showed a peak between August and October in 2016, 2018 and 2023 (Figure 3B). For *C. parvum*, the number of cases was also low from January until June (≤ 15 cases/month) and slightly higher from July to September but never exceeded 30 cases per month (Figure 3C). The peaks in *Cryptosporidium* spp. infections observed in 2016, 2018 and 2023 were primarily driven by *C. hominis*.

### Subtyping of *Cryptosporidium hominis*

We *gp60* subtyped 207 samples from 2019 to 2024 and included data about  313 samples from published datasets from 2003–2005 [15] and 2012 [16] (Table 1). A total of 23 subtypes were identified: 18 in 2019–2024 and nine in 2003–2005 [15] and 2012 [16]. In 2019–2024, IdA16 was the most common subtype (43/207; 21%) and in 2003–2005 [15] and 2012 [16], subtype IbA10G2 dominated (283/313; 90%).

**Table 1:** Subtypes of *Cryptosporidium hominis* in samples from patients with laboratory-confirmed cryptosporidiosis, the Netherlands, 2003–2024 (n = 520)^a, b^

*gp60* subtype	2003–2005 (n = 68) [15]		2012 (n = 245) [16]		2019 (n = 38)		2020 (n = 13)		2022 (n = 64)		2023 (n = 70)		2024 (n = 22)	
	n	%	n	%	n	%	n	%	n	%	n	%	n	%
Ia (n = 40)														
IaA12R3	0		0		0		0		2	3	8	11	0	
IaA13R3			0						0		1	1	0	
IaA14R3			21	8.6					0		0		3	14
IaA15			1	0.4					0		0		0	
IaA15R3			0						1	2	0		0	
IaA18R3			0						1	2	0		0	
IaA24G1R1			1	0.4					0		0		0	
IaA24R3			0						0		1	1	0	
Ib (n = 373)														
IbA9G3a	0		0		0		0		5	8	8	11	0	
IbA10G2	65	96	218	89	19	50	7	54	0		2	3	5	23
IbA12G3	0		0		19	50	6	46	1	2	0		0	
IbA13G2	0		0		0		0		0		1	1	0	
IbA13G3	0		0		0		0		7	11	9	13	1	5
Ic (n = 1)														
IcA5G3R2	1	1	0		0		0		0		0		0	
Id (n = 49)														
IdA13	0		0		0		0		0		1	1	0	
IdA14	1	1							0		0		0	
IdA15G1	0								0		0		1	5
IdA16	0								21	33	15	21	7	32
IdA17	1	1							1	2	0		0	
IdA24	0								0		0		1	5
Ie (n = 40)														
IeA11G3T3	0		3	1.2	0		0		17	27	16	23	4	18
If (n = 17)														
IfA12G1	0		1	0.4	0		0		0		0		0	
IfA12G1R5	0		0						8	13	8	11		

^a^ Data from three medical microbiology laboratories.

^b^ *C. hominis* was not detected in 2021.

To explore if the peak in *C. hominis* infections in 2023 might be associated with changes in circulating *C. hominis* subtypes, 70 of the 444 samples from 2023 were subtyped. We identified 11 distinct subtypes, with IeA11G3T3 (16/70; 23%) and IdA16 (15/70; 21%) the most common, followed by IbA13G3 (9/70; 13%), IbA9G3a (8/70; 11%) and IfA12G1R5 (8/70; 11%) (Table 1). Additional *C. hominis* samples from 2019 to 2022 were *gp60* subtyped to assess if the increase in diversity began after the absence of *C. hominis* in 2021. In 2019 and 2020, only two subtypes were detected: IbA10G2 (19/38; 7/13) and IbA12G3 (19/38; 6/13). However, in 2022, 10 distinct subtypes were identified, with subtype IdA16 (21/64; 33%) and IeA11G3T3 (17/64; 27%) dominating. To assess if the increased *gp60* diversity persisted, additional *C. hominis* samples from 2024 were typed. In 2024, seven *gp60* subtypes were detected in 22 samples, IdA16 being the most common (7/22). Between 2019 and 2020, the subtype diversity of *C. hominis* was low with a Simpson Diversity Index of 0.50, but in 2022–2024, the diversity increased significantly to 0.83 (permutation test p value < 0.001).

### Association between *Cryptosporidium hominis* and precipitation or temperature

To explore if fluctuations in temperature or precipitation could explain the peaks in *C. hominis* infections, we performed a Pearson’s correlation test. There was no statistically significant association between mean weekly temperature (ρ = 0.18; p value = 0.0758 at a time lag of 6 weeks) and precipitation (ρ = −0.02; p value = 0.878 at a time lag of 6 weeks) and the number of *C. hominis* when including only weeks between July and September (week 27–40) (Table 2). Including all weeks of the year (week 1–52) suggested a weak association of mean temperature with the number of *C. hominis* cases, with the strongest correlation at a time lag of 6 weeks (ρ = 0.37; p value < 0.001), but no association with precipitation (ρ = 0.10; p value = 0.060 at a time lag of 0 weeks).

**Table 2:** Association between mean weekly temperature and precipitation and the number of patients with laboratory-confirmed infection with *Cryptosporidium hominis*, the Netherlands 2016–2024 (n = 1,166)^a^

Time lag (weeks)	Week 27–40 (July–September)				Week 1–52			
	Mean precipitation (mm)		Mean temperature (°C)		Mean precipitation (mm)		Mean temperature (°C)	
	ρ	p value	ρ	p value	ρ	p value	ρ	p value
0	−0.11	0.290	−0.22	0.028	0.10	0.060	0.10	0.049
1	−0.07	0.495	−0.12	0.257	0.08	0.114	0.15	0.004
2	−0.14	0.169	−0.07	0.496	0.02	0.637	0.20	< 0.001
3	−0.10	0.340	0.06	0.577	0.03	0.524	0.26	< 0.001
4	−0.10	0.322	0.09	0.371	0.01	0.814	0.29	< 0.001
5	−0.03	0.799	0.17	0.104	0.04	0.497	0.34	< 0.001
6	−0.02	0.183	0.18	0.076	0.03	0.516	0.37	< 0.001

^a^ Data from three medical microbiology laboratories.

Pearson’s correlation test was used to determine the relationship between mean weekly temperature and precipitation (in mm) and the number of patients with *C. hominis* infection.

## Discussion

In the late summer of 2023, a peak of *Cryptosporidium* spp. infections was observed in the Netherlands. The peak was driven by multiple distinct *gp60* subtypes of *C. hominis*. In 2024, a similar peak in infections was not observed, but the diversity in subtypes persisted.

In the Netherlands, *C. hominis* infections are an important cause of the annual fluctuations in cryptosporidiosis. In most European countries, *C. parvum* is the dominant species causing cryptosporidiosis. The reported proportion of *C. hominis* cases differs per country, with relatively lower proportions reported for Sweden (2018–2022: 3.7%), Norway (2022–2024: 18.9%), Scotland (2018–2020: 14%) and France (2017–2019: 24%), and higher proportions for Spain (2023: 94.3%) and the Netherlands (2016–2024: 55%) [5,12,24-27]. Exposures more commonly associated with *C. hominis* are the use of (inflatable) pools, international travel or increased contact with or care for young children [6,12,17,28]. Collecting patient information on these factors associated with *C. hominis* is a key to understanding why the proportion of *C. hominis* is high in the Netherlands compared with other European countries and could help inform potential public health interventions.

The annual trends in *Cryptosporidium* spp. infections in the Netherlands with a peak in 2023 and low numbers of infections in 2020–2022 and 2024, are very similar to those described for other European countries [5,12,13,29]. The substantial decline in *C. hominis* cases and the moderate decrease in *C. parvum* infections during 2021 and 2022 is most plausibly linked to the COVID-19 restrictions implemented across Europe, including the Netherlands, as also demonstrated for other European countries and New Zealand [7,13,24,26,30]. This differential impact likely reflects the predominant transmission routes of each species, *C. hominis* is predominantly transmitted via (in)direct human-to-human contact, whereas *C. parvum* is associated with both human and animal sources. The reduction in international travel has been suggested as an explanation for the strong decline in *C. hominis* cases specifically, as *C. hominis* infections were suggested to be seeded by international travel [7,24,26,28,30]. However, other public health interventions, such as closing of swimming pools and daycares, and enhanced hand hygiene, probably also contributed to the decline of both *C. hominis* and *C. parvum* infections [7,26,30]. Unfortunately, a previous risk factor study for cryptosporidiosis conducted in the Netherlands from 2013 to 2016 excluded participants with a history of international travel, thus this study could not identify the role of international travel in *C. hominis* infections in the Netherlands [17].

In contrast to several but not all European countries, but consistent with findings from 2003 to 2005 [5,13,15,25], no spring peak in *C. parvum* infections was observed in the Netherlands in 2016–2024. Despite the absence of a peak, *C. parvum* is responsible for 45% of cryptosporidiosis cases in the Netherlands. A source attribution study using molecular epidemiology data from both human and animal samples could help estimate the role of anthroponotic and zoonotic transmission routes. Although not all *C. parvum gp60* subtypes are zoonotic (e.g. IIc), visiting a farm has been identified as a risk factor. Furthermore, subtyping has demonstrated an overlap in *gp60* subtypes between human and cattle isolates [15,17], suggesting ongoing transmission between animals and humans without a seasonal peak in the Netherlands.

*Cryptosporidium hominis* IbA10G2 has been the dominant circulating *gp60* subtype in Europe for a long time, including the Netherlands [6,15,16]. Since the period in which COVID-19 restrictions were implemented (2020–2021), the circulating subtype in the Netherlands has shifted towards a diverse set of subtypes. Identical shifts in circulating *C. hominis gp60* subtypes away from IbA10G2 have been identified in Norway, Spain and Scotland [12,25,26]. In Spain (62%) and Norway (22%), IfA12G1R5 was identified as (one of) the dominant circulating subtype [12,25], which was marked as hyper-transmissible in the US [31]. In the Netherlands, in 2022 and 2023, IfA12G1R5 was only identified in eight samples and was absent among the 22 samples received in 2024. The *C. hominis* subtypes identified in Scotland (IeA11G3T3, IdA16 and IbA9G3) and Norway (IeA11G3T3 and IbA9G3) were also among the most identified subtypes in the Netherlands [25,26]. Additional molecular typing and whole genome sequencing of *C. hominis* samples collected in 2023 can help identify the sources of these new *gp60* subtypes and identify genes introduced in the population that confer increased transmissibility such as the invasion-associated mucin-like glycoproteins [31]. Restrictions due to COVID-19 seem to have halted the transmission of the locally dominant *C. hominis gp60* subtype IbA10G2 in Europe due to its dependency on human-to-human transmission. After COVID-19 restrictions were removed, these novel *C. hominis gp60* subtypes may have been introduced in Europe via international travel to countries outside of Europe [26]. The introduced *gp60* subtypes vary between countries and started circulating locally, as well as within Europe.

Several studies have found associations between meteorological variables and the incidence or outbreaks of *Cryptosporidium* spp. infections [18]. In some high-income countries, increases in cryptosporidiosis incidence were associated with an increased temperature and precipitation, although the type of association differed per country [18]. In our analysis, we observed a weak association between the number of *C. hominis* cases and temperature, gradually increasing over longer time lags up to 6 weeks. The latter questions the plausibility of the association, since *Cryptosporidium* spp. have an average incubation period of 7 days, and the strongest correlation would therefore be expected at a shorter time lag. Behavioural factors may explain the observed association as well. During the summer, many people travel abroad and engage in recreational water activities, thereby increasing their exposure to cryptosporidiosis [6]. Therefore, despite the described impact of temperature on oocysts survival and the contribution of rainfall to oocysts dispersal [32,33], these meteorological factors alone cannot explain why certain years show peaks in cryptosporidiosis incidence in the Netherlands.

The complex immunological mechanisms underlying the clearance of *Cryptosporidium* spp. and the development of sterilising immunity are multifactorial and not yet fully understood [34]. Primary infection induces both humoral and cellular immune responses but does not confer sterilising immunity. Instead, it results in reduced shedding of oocysts and milder disease upon reinfection [34-36]. The low incidence of cryptosporidiosis from 2020 to 2022 may have left a larger proportion of the population naïve for *Cryptosporidium* spp. When these naïve individuals become infected, they tend to shed higher numbers of oocysts, which can increase transmission within the population. We speculate that this enlarged group of susceptible individuals may have contributed to the observed peak in *C. hominis* in 2023.

Our study has several limitations regarding the generalisability of our results to the whole country, the restricted patient data and limitations in subtyping. Firstly, it is difficult to establish the true incidence of cryptosporidiosis in the Netherlands due to its non-notifiable status and the absence of a nationwide surveillance system, which makes it difficult to assess how representative our study is for the whole country. Secondly, the collected patient data lacked key information on potential risk factors such as international travel, engagement in recreational water and increased contact with children. The absence of travel history data is of relevance, as travel-associated cases should have been excluded from the analysis examining the association between *Cryptosporidium* cases and meteorological variables. Only domestically acquired cases are expected to be influenced by local meteorological factors. A previous Dutch study in 2013–2016 indicated that 27% (228/837) of laboratory-confirmed *Cryptosporidium* cases were travel-associated [17]. However, our study included the COVID-19 pandemic period, thus the percentage of travel-associated cases in the current study is likely much lower. Nevertheless, the lack of information on exposure to potential risk factors limits our ability to identify specific transmission routes. Thirdly, there were methodological limitations related to *gp60* subtyping. In certain years, the number of available samples was limited, or only a selection could be analysed, therefore, rarer *C. hominis gp60* subtypes were likely missed. Furthermore, we chose to focus our *gp60* subtyping efforts on *C. hominis* because it was responsible for the peak in infections in 2023, thereby not providing subtyping information on *C. parvum* and other *Cryptosporidium* species. Lastly, a better insight in infection dynamics could be obtained using more advanced subtyping methods such as multilocus sequence typing, multilocus variable-number tandem repeat analysis or whole genome sequencing.

To improve monitoring and enhance understanding of *Cryptosporidium* spp. population dynamics, the integration of additional genotyping or whole genome sequencing is essential. Enhanced molecular surveillance will allow for early detection of emerging strains or introduction of genes associated with increased virulence. Moreover, the increased molecular resolution compared with *gp60* could allow for a more effective outbreak response. Lastly, international data sharing and collaboration will be important to monitor trends, identify sources and respond to potential cross-border *Cryptosporidium* spp. outbreaks or peaks in incidences.

## Conclusion

The annual fluctuations in Dutch cryptosporidiosis cases including the peak in 2023 were caused by *C. hominis*. These fluctuations did not seem to be strongly associated with precipitation or temperature. During the COVID-19 pandemic, especially in 2021, *C. hominis* transmission was halted almost completely, while the *C. parvum* transmission appeared to be less affected. Since 2022, patient numbers and subtype diversity of *C. hominis* has increased in the Netherlands. Continuous surveillance, including exposure to risk factors and molecular typing, is key to better understanding of the underlying causes of *Cryptosporidium* spp. infections.

## Data Availability

Data are available on reasonable request to the authors. Restrictions apply to the availability of data linked to patient information. All gp60 sequencing data is publicly available via GenBank PX853406–PX853612.
